# On-Line Core Losses Determination in ACSR Conductors for DLR Applications

**DOI:** 10.3390/ma15176143

**Published:** 2022-09-04

**Authors:** Jordi-Roger Riba, Yuming Liu, Manuel Moreno-Eguilaz, Josep Sanllehí

**Affiliations:** 1 Electrical Engineering Department, Universitat Politècnica de Catalunya, Rambla Sant Nebridi 22, 08222 Terrassa, Spain; 2 SBI Connectors, Albert Einstein, 5, 08635 Sant Esteve Sesrovires, Spain

**Keywords:** core losses, aluminum conductor steel-reinforced, ac resistance, dynamic line rating

## Abstract

Dynamic line rating (DLR) is a method that focuses on dynamically determining the maximum allowable current of power lines, while ensuring they operate within safe limits. DLR needs to monitor the temperature and current of the line in real-time, as well as the weather variables in the surroundings of the power line. DLR approaches also require determining the AC resistance of the power line conductors, which is a key parameter that enables it to determine Joule and core losses. This paper presents an approach for an on-line alternating current (AC) resistance estimation of aluminum conductor steel-reinforced (ACSR) conductors to determine the DLR capability of such conductors from real-time conductor and meteorological parameter measurements. For this purpose, conductors with one, two and three layers of aluminum strands are analyzed in detail. Based on the experimental results presented in this paper, two possible approaches are proposed.

## 1. Introduction

Today, renewable power generation sources are reinforcing distribution networks. The growing demand for electricity is forcing system operators to take full advantage of the maximum capacity of existing power lines [1]. However, the maximum allowable temperature of the conductor must not be exceeded, as it defines the current carrying capacity of the conductor [2]. The ampacity or maximum allowable loading current is established by the maximum permissible temperature of the conductor [3]. Temperature has a great influence on the mechanical sag of overhead transmission lines [4,5], which is a key parameter for power system operation [6]. Overhead power transmission lines are generally operated below their static line rating (SLR). The SLR is calculated when the conductor operates at the maximum allowable temperature in conservative weather conditions. Contrarily, dynamic line rating (DLR) determines the actual ampacity when conductors are operating at the maximum allowable temperature under actual weather conditions [7]. DLR can be useful for the integration of intermittent renewable energy sources into existing power networks [8]. Intermittent renewable energy sources (IRES) cannot provide constant additional power, making it difficult for system operators to justify the investment required to expand current transmission lines [9]. Dynamic line rating (DLR) allows for the maximum utilization of the conductor, that is, to operate at the maximum ampacity or current that it can withstand without exceeding the upper allowed temperature of the conductor [10]. Although there are different approaches to DLR, the most common approach is to measure weather variables, line current and temperature using dedicated sensors [11]. These measured variables are used to feed mathematical models of conductors, such as the Cigré TB207 [12] and IEEE [13] models, from which the maximum conductor rating is dynamically obtained. Results obtained are significantly dependent on weather conditions, including ambient temperature, wind speed and direction, or solar radiation, although wind speed significantly affects the resulting ampacity [14,15].

The alternating current resistance (*R_ac_*) of the conductor is a key parameter which allows the ampacity or maximum current capacity of power conductors to be accurately estimated. This can increase their effective capability [16], since Joule and core losses represent the main heat source of the conductor, and can be determined from the alternating current (AC) resistance *R_ac_* [17,18]. Line current, along with ac resistance, are used in some DLR approaches to estimate the effective wind speed [18]. Therefore, DLR enables the development of more accurate power flow analysis approaches if the ac resistance, which changes with temperature, is measured in real time [19].

**Figure 1:**
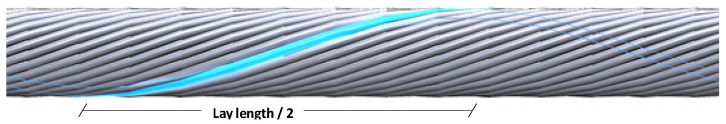
Stranded conductor and lay length.

Stranded aluminum conductor steel-reinforced (ACSR) conductors have been applied for over 100 years in overhead power lines to transmit electrical power at high voltage [20]. ACSR conductors have multiple layers of aluminum [21] and galvanized steel strands. The aluminum wires are wound helically around the core formed by the galvanized steel strands. Adjacent aluminum layers are wound in opposite directions around the steel core [22]. Whereas the steel core provides the mechanical strength, high-conductivity aluminum strands provide the current path [23], carrying approximately 98% of the electric current [24]. Electric current tends to spiral along the aluminum strands because the contact resistance between adjacent strands is much greater than the internal resistance of the strands, producing an axial component of the magnetic field. The axial AC magnetic flux causes eddy currents and hysteresis losses in the core, and forces the current density in the aluminum layers to redistribute to a greater or lesser extent, depending on the strength of the magnetic field in the core, and thus, on the intensity of the electric current and the lay length of the aluminum layers [25]. The lay length is the distance the wire requires to make a complete revolution around the diameter of the conductor, as shown in Figure 1. Therefore, due to magnetic induction, the steel core increases the AC resistance of the conductor [23].

Due to the opposite stranding directions of adjacent aluminum layers, the axial components of the magnetic field generated by the currents from adjacent layers are in opposite directions, which reduces the total magnetic field. The greatest cancellation of the magnetic field occurs in conductors with two aluminum layers, this effect being less marked in conductors with an odd number of aluminum layers than in conductors with an even number of layers [23,25,26]. Therefore, ACSR conductors with an even number of aluminum layers have lower magnetic core losses [22]. Under AC supply, the axial component of the magnetic field generates eddy currents and hysteresis losses, and modifies the current distribution between the layers. This effect, due to the magnetic coupling of the current in the aluminum layers through the steel core [3], is known as the transformer effect. The magnitude of the axial magnetic flux largely determines the intensity of the transformer effect, so it varies with the intensity of the electric current and the magnetic permeability of the steel core, which in turn depends on the tensile stress of the core and the temperature [27].

The non-uniformity of the current density in the different layers of aluminum due to the transformer effect is greatest for three-layer ACSR conductors [3]. Experimental results performed on a three-layer ACSR conductor show that the current density in the central layer is higher than in the other layers, resulting in a higher *R_ac_*/*R_dc_* ratio [28], *R_dc_* being the direct current (DC) resistance. This is mainly due to the transformer effect [26,27] or current redistribution among the aluminum layers, and only partially due (to a lesser extent) to the eddy currents and hysteresis losses that occur in the steel core [28].

This paper presents an approach for an on-line estimation of the AC resistance of ACSR conductors to determine their DLR capacity from real-time conductor measurements and weather parameters, thus presenting several novel findings and making unique contributions in this area. Joule and core losses are determined from an on-line measurement of the AC resistance, which, due to its low value, makes its on-line measurement under AC supply a challenging task. The approach proposed here to estimate the AC resistance can be applied to ACSR conductors with any number of aluminum layers, while considering its dependence on the line current. This is a novelty in this work, since most of the current DLR approaches do not take into account the change in the AC resistance of ACSR conductors with the current level, so they can lead to inaccurate estimates of the AC resistance, and thus of the DLR ampacity. To this end, two new methods are proposed to determine the AC resistance. The first one, which is more accurate, is based on measuring temperature, current, voltage drop and the phase shift between voltage drop and current over a short length of conductor. The second method only requires measuring the temperature and current through the conductor, thus avoiding measuring the voltage drop and simplifying installation and measurement requirements. The proposal presented here is in line with the current trend of operating power lines at their maximum instantaneous capacity, since it allows the current rating to be adapted to existing weather conditions in real-time. In addition, it is simple to apply, requires a low computational burden, and is compatible with the Internet of Things (IoT), one of the crucial technologies that improves smartness in many industrial, power line and smart grid applications [29,30,31], whose basic function is to connect objects [32], including intercommunicating sensors [33], actuators and other smart technologies [34].

The experimental results presented in this paper show that the two proposed approaches are suitable for an on-line determination of the AC resistance of ACSR conductors to apply DLR strategies, so that it is possible to take advantage of the full transmission capacity of ACSR-based power lines. The accuracy of the proposed approaches has been evaluated by analyzing different ACSR conductors, including single-, two- and three-layer conductors in different working conditions, covering a wide range of operations in different weather conditions.

This paper is structured as follows: Section 2 describes the theoretical background, including how to experimentally determine the AC resistance of ACSR conductors, the equations required to estimate power losses in ACSR conductors, the transient thermal balance equation for ACSR conductors and the calculation of the DLR ampacity; Section 3 describes the experimental setup used in this paper, including the electrical loop, the analyzed conductors, the high-current power transformer, the sensors used and the measuring devices; Section 4 shows the experimental results obtained with the different conductors and discusses the results attained; and finally, Section 5 concludes the paper.

## 2. Theoretical Background

### 2.1. AC Resistance and Reactance of ACSR Conductors

The AC resistance of a conductor is affected by the current density distribution, the internal temperature and the magnetic properties of the steel core, with the two last parameters being affected by the intensity of the electric current. According to [35], the AC resistance of ACSR conductors can be expressed as the sum of three terms, i.e., the DC resistance, the resistance term due to eddy currents, and the term due to hysteresis losses. Core losses (hysteresis and eddy current losses) as well as any temperature rises also increase the effective AC resistance of the conductor [3,20,36]. The combined action of eddy currents, hysteresis and the transformer effect raises the AC resistance *R_ac_* of the conductor above the DC value *R_dc_* at the same temperature. Higher values of the *R_ac_*/*R_dc_* ratio increase the energy losses in the conductor [20].

Since the cancellation of the axial component of the magnetic field is less effective in conductors with an odd number of aluminum layers, these conductors will exhibit higher resistance ratios *R_ac_/R_dc_*. The highest resistance ratio is expected in conductors with a single layer of aluminum, whereas the lowest corresponds to conductors with two layers [3]. The internal inductance of ACSR conductors increases with current up to a maximum value, where the steel core becomes magnetically saturated [36], and then any further increase in current reduces the internal inductance [3] due to the decrease of the magnetic permeability.

According to the international standard IEC 60287-1-1 [37], the AC resistance can be obtained from the DC resistance as:

(1) $$R_{ac}=R_{dc}(1+y_{s}+y_{p})\;[\Omega /m]$$

where *y_s_* and *y_p_* are, respectively, the skin and proximity effect factors. The effect of temperature is considered as:

(2) $$R_{dc}=R_{0}[1+\alpha_{20}(T-20)]\;[\Omega /m]$$

where *T* is the operating conductor temperature, *R*_0_ the DC resistance measured at 20 °C and *α*_20_ is the temperature coefficient at 20 °C.

In most ACSR applications, the skin effect is negligible compared to the transformer effect, but in applications requiring low currents, large diameter conductors or high frequencies, this difference is reduced. The proximity effect is also negligible when distances between adjacent conductors greater than ten diameters are assumed [20]. Although (1) is simple to apply, it only applies to nonferromagnetic conductors up to 5 kV [37], and it does not consider important parameters such as those related to the stranding, including the direction and lay length, as it considers the stranded conductor to be a solid conductor [16,38].

The DC resistance of the conductor only depends on the temperature of the conductor, but the AC resistance also depends on the current [3]. According to [25], the AC resistance of an isothermal ACSR conductor increases with current following a sigmoidal change due to the change in magnetic field strength and the consequent increase in core losses.

There is no explicit equation to determine the internal inductance of an ACSR conductor under DC supply. Several authors have proposed formulas for AC supply, although they are not exact, since it is difficult to model the complex behavior of the magnetic flux in the steel core. It is known that the per-unit length of the internal AC inductance of ACSR conductors also increases with current following a sigmoidal curve up to a maximum value corresponding to the magnetic saturation of the steel core, and then, any further current increases reduce the inductance [25]. The goodness of the electrical contacts between aluminum wires in the same layer and between wires in adjacent layers changes with the tension and degree of oxidation in the conductor. Also, conductors are not isothermal because they exhibit both radial and sometimes axial temperature gradients [3]. Therefore, due to the nonlinearity and complexity of these effects, the most effective way to determine the AC resistance and the internal reactance or inductance of stranded ACSR conductors is from experimental measurements.

By measuring the instantaneous values of the voltage drop per unit length Δ*V*, the electric current *I* and the phase shift *φ* between them, it is possible to determine the per unit length values of the impedance [39], AC resistance *R_ac_* and reactance *X* of the conductor expressed in Ω/m as [3,40]:

(3) $$Z=\frac{\Delta V}{I}\;[\Omega /m]$$

(4) $$R_{ac}=\frac{\Delta V}{I}\cdot \cos\varphi \;[\Omega /m]$$

(5) $$X=\frac{\Delta V}{I}\cdot \sin\varphi \;[\Omega /m]$$

The next equation describes the dependence of *R_ac_* with temperature:

(6) $$R_{ac,T}=R_{ac,0}[1+\alpha_{ac}(T-T_{0})]\;[\Omega /m]$$

where *R_ac_*_,0_ is the AC value of the resistance at a given temperature *T*_0_, usually 20 °C, and *α_ac_* is the temperature coefficient of the AC resistance. Note that *R_ac_*_,0_ is a measured value, which already includes the saturation effect, as proved in [19].

### 2.2. Power Losses in ACSR Conductors

Core losses take into account the effect of eddy currents and hysteresis in the steel core [3]. These losses, along with current redistribution and the effect of temperature, are key factors determining the AC resistance of ACSR conductors [41]. Eddy current and hysteresis losses increase with the square of the strength of the magnetic flux density and with increasing values of temperature for a constant strength of the magnetic field *H*. However, they tend to decrease as the resistivity of the steel core material increases, and thus, as the temperature rises [20].

According to [17,18,42], the losses due to Joule (*P_J_*), core (*P_M_*) and redistribution (*P_redi_*_s_, transformer effect) effects can be expressed as follows:

*P_J_* + *P_M_* + *P_redis_* = *I*^2^*R_ac_* (7)

Note that (7) is in agreement with the method for measuring power losses detailed in IEEE Std. 2772 [43] to measure the power loss of overhead conductors under real simulated laboratory conditions:

*P_loss_* = *P_J_* + *P_M_* + *P_redis_* = Δ*VI*cos*φ* (8)

### 2.3. Transient Thermal Balance Equation for DLR Calculation

The heat balance equation, which must always be satisfied, is the basis for implementing DLR approaches for ACSR conductors [18]. It states that the sum of the ohmic (*P_J_* + *P_M_* + *P_redi_*_s_) and solar heat gain must equal the heat loss by convection and radiation [44].

According to [12], the unsteady-state equation that describes the transient thermal balance of an ACSR conductor can be expressed as:

(9) $$P_{J}+P_{M}+P_{redis}+P_{S}-P_{C}-P_{R}=mc\frac{dT}{dt}\;[W/m]$$

*P_J_*, *P_M_*, *P_redis_* and *P_S_* being the per unit length heat gain terms of the conductor (Joule, magnetic/core, transformer effect and solar heating terms, respectively, in W/m). *P_C_* and *P_R_* are the per unit length heat loss terms (convective and radiative loss terms, respectively, in W/m), *m* is the per unit length mass of the conductor in kg/m, *c* is the specific heat capacity of the conductor expressed in J/(kg °C), *T* is the average conductor temperature expressed in °C, and *t* is the time in s. The heat capacity *c* of the ACSR conductor is calculated as the weighted average of the iron strands in the core and the aluminum strands, and can be expressed as:

(10) $$c(T)=\frac{m_{Al}c_{Al,20{}^{\circ}C}[1+\beta_{Al}(T-20)]+m_{steel}c_{steel,20{}^{\circ}C}[1+\beta_{steel}(T-20)]}{m_{Al,20{}^{\circ}C}+m_{steel,20{}^{\circ}C}}\;[J/(\mathrm{kg}{}^{\circ}C)]$$

where *c_x_* is the specific heat capacity of element *x* (*Al* = aluminum or steel), *m_x_* is its mass per unit length, and *β* is the temperature coefficient of the heat capacity, whose values can be found in [10].

According to [10,18], convective heat losses are calculated as:

(11) $$P_{C}=\pi [0.042+3.6\cdot 10^{-5}(T_{a}+T)](T-T_{a})Nu\;[W/m]$$

where *Nu* is the Nusselt number, which can be calculated as detailed in [18] once the conductor diameter, surface roughness, local wind speed, local temperature, relative density and kinematic viscosity of the air are known.

The heat gain by solar radiation is obtained from the global solar radiation *S* (W/m^2^) as [12]:

*P_S_* = *α_s_SD* [W/m] (12)

where *D* [m] is the outer conductor diameter and *α_s_* is the dimensionless solar absorptivity of the conductor surface, where its value is often assumed to be 0.5 [45].

Heat losses due to radiation are calculated as [12]:

*P_R_ = πεDσ_B_*[(*T* + 273)^4^ − (*T_a_* + 273)^4^] [W/m] (13)

where *ε* is the emissivity factor of the surface of the conductor (its value is often assumed to be 0.5 [2,45]) and *σ_Β_* is the Stefan-Boltzmann constant.

The DLR ampacity *I_max_* is calculated at the maximum allowable temperature of the conductor, assuming that the temperature of the conductor is in thermal equilibrium, resulting in [10,46]:

(14) $$I_{\max}={\left(\frac{P_{C}(T_{\max})+P_{R}(T_{\max})-P_{S}}{R_{ac}(T_{\max})}\right)}^{1/2}\;[A]$$

From (14), it is seen that the AC resistance plays a key role in determining the DLR ampacity, so an accurate estimate is required for this purpose.

## 3. Experimental Setup

This section describes the experimental devices and materials used in the laboratory experiments, including the ACSR conductors, sensors and measuring devices required to determine the AC resistance of the studied conductors.

### 3.1. The Analyzed Single-, Two- and Three-Layer ACSR Conductors

As explained in the introduction, ACSR conductors with one, two or three layers have different behaviors due to the interaction of the axial magnetic flux generated by the different layers of aluminum strands with the steel core, thus increasing the AC resistance of the conductor. Since the adjacent aluminum layers are stranded in opposite directions, the axial component of the resulting magnetic field is reduced. Therefore, this cancellation effect is greatest in two-layer ACSR conductors, while it is the least impactful in three-layer conductors, and not present at all in single-layer conductors. Due to the effect of the steel core, the AC resistance of the conductor is expected to depend not only on the temperature of the conductor but also on the intensity of the current flowing through the conductor. However, due to the partial cancellation of the axial component of the magnetic flux generated by layers of aluminum strands wound in opposite directions, the AC resistance of two-layer ACSR conductors should be almost independent of current intensity, while in the case of three-layer ACSR conductors, this dependency should be very low. It should be at the maximum in single-layer ACSR conductors.

This work analyzes the behavior of the three-layer 550-AL1/71-ST1A ACSR conductor (HAASE Gesellschaft mbh, Graaz, Austria), and the two-layer 135-AL1/22-ST1A conductor (EMTA Kablo, Istanbul, Turkey), whose main parameters are summarized in Table 1.

Next, a single-layer ACSR conductor was made from the 135-AL1/22-ST1A two-layer conductor by carefully removing the outer layer of aluminum strands.

The three-layer conductor includes 7 steel strands that form the core, and three successive layers with 12, 18 and 24 aluminum strands, respectively, thus being 7/54.

The two-layer conductor includes 7 steel strands in the core and two layers with 10 and 16 aluminum strands, respectively, being 7/26. The single-layer conductor is 7/10.

**Figure 2:**
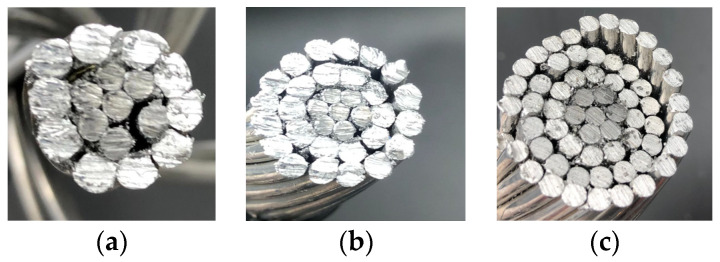
Cross section of the analyzed ACSR conductors. (**a**) Single-layer 7/10 conductor. (**b**) Two-layers 7/26 conductor. (**c**) Three-layer 7/54 conductor.

Figure 2 shows the geometry of the analyzed three-, two- and single-layer ACSR conductors.

### 3.2. The High-Current Transformer Used to Test the Conductors

**Figure 3:**
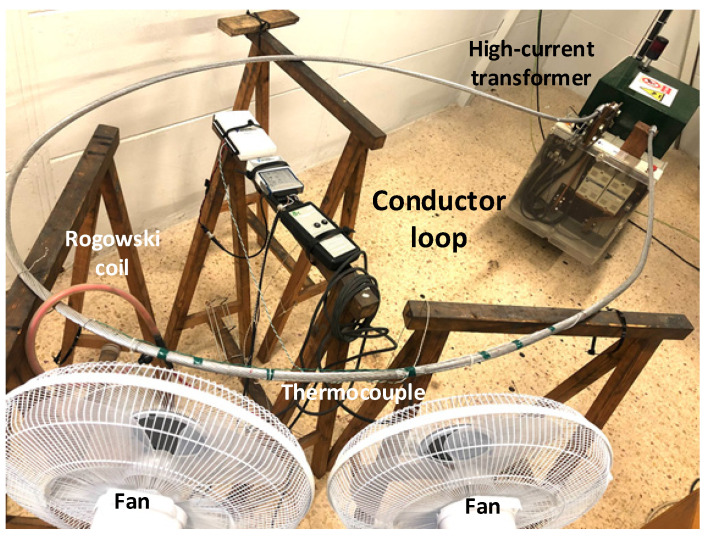
Experimental loop for testing the different ACSR conductors.

To determine the AC resistance of the conductors, they were tested in the laboratory under different operating conditions. For this end, a variable high-current transformer (10 kVA, 380 V/4 V, output current 0–2.5 kA, Transcir, Montcada i Reixac, Spain) was used to generate the current required to test the conductors. The analyzed ACSR conductors were connected to the high-current transformer forming a low impedance circular loop, as shown in Figure 3. To meet the requirements of the Cigré TB345 [3], the length of the conductor was 5.5 m.

It is known that wind speed has a stronger impact in terms of DLR rating than ambient temperature or solar radiation [9], so the effect of wind speed was studied in this paper. As shown in Figure 3, two SF 0147 fans (variable speed, 50 W, Orbegozo, Murcia, Spain) were used to simulate variable wind speed conditions. It should be noted that in the experiments, a constant wind flow of 5 m/s was applied, which was measured with a Pen 850021 anemometer (0.4–30 m/s measuring range, resolution of 0.1 m/s, accuracy of 3% full scale, Sper Scientific, Scottsdale, AZ, USA) during the entirety of the experiment under wind conditions.

### 3.3. Measuring Devices

In experimental tests involving the loops of ACSR conductors, the current flowing in the loop, the voltage drop over one meter length of the conductor and the phase shift between the voltage drop and current was measured.

The current flowing through the test loop was measured by means of a CWT500LFxB Rogowski coil (sensitivity = 0.06 mV/A, PEM, Nottingham, UK), which was connected to an NI USB-6210 data acquisition system (National Instruments, Dallas, TX, USA), with a current accuracy of ±1%.

A T-type thermocouple was used to measure the surface temperature of the conductor. It was connected to a NI-9211 thermocouple input module (±1 °C, National Instruments, Dallas, TX, USA).

Acquisitions made with the NI-9211 thermocouple input module and the NI USB-6210 data acquisition system were synchronized using Python code programmed by the authors of this work.

An 850021 RH Pen anemometer was used to measure the wind speed (measuring range 0.4–30 m/s, 0.1 m/s resolution, 3% full scale accuracy, Sper Scientific, Scottsdale, AZ, USA).

## 4. Experimental Results

This section presents the experimental tests carried out with the three ACSR conductors described in Section 3, which were performed in the AMBER high-current laboratory facilities of the of the Universitat Politècnica de Catalunya.

### 4.1. Results Obtained with a Single-Layer ACSR Conductor

The first test was conducted with a single-layer conductor, whose rated current was about 220 A. The conductor was heated from room temperature by applying the rated current until reaching the equilibrium temperature, which was about 77 °C. Conventional ACSR conductors usually operate below 90 °C [24] except when operating under emergency contingency conditions [19].

**Figure 4:**
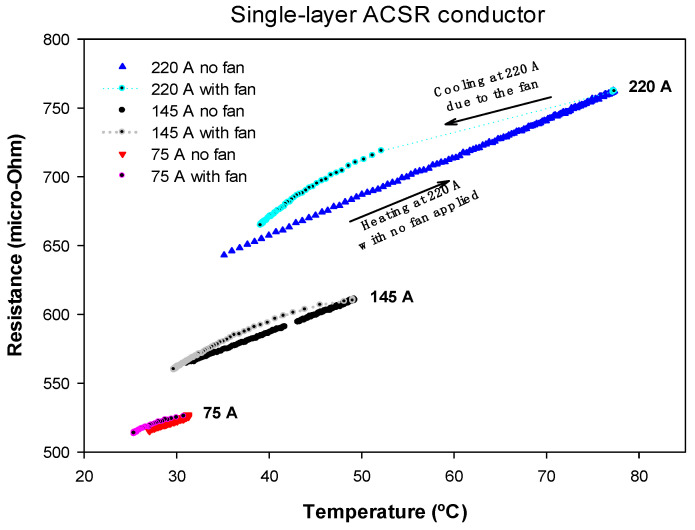
Single-layer ACSR conductor. *R_ac_* versus temperature measured for heating-cooling cycles at 220 A, 145 A and 75 A.

Then, by forcing it to pass the same amount of current, the conductor was cooled with the help of fans. During both parts of this cycle, the temperature of the conductor, the voltage drop and the current through the conductor were measured. From these measurements, the phase shift between the voltage drop and the current was calculated, and the AC resistance was obtained from (4). This test was repeated with currents of 145 A and 75 A. Figure 4 shows the results of the AC resistance as a function of the conductor temperature.

Results presented in Figure 4 clearly show the effect of the core on the AC resistance of the single-layer ACSR conductor. These results proved that the AC resistance of a single-layer ACSR conductor depended not only on temperature but also on the current flowing through the conductor. Results in Figure 4 also show that, in the case of strong wind, the measured surface temperature differs more from that of the interior. Therefore, for a given conductor surface temperature, the apparent AC resistance measured in strong wind is higher than when measured in no wind because of the higher temperature difference between the interior and the surface of the conductor in strong wind conditions (increased radial temperature gradient). However, this difference is always below 5%.

### 4.2. Results Obtained with a Two-Layer ACSR Conductor

The second test was carried out with a two-layer conductor, whose rated current was about 430 A. Due to the opposite stranding directions of the two layers, the axial component of the magnetic flux is almost cancelled. Therefore, the AC resistance must be almost independent of the current intensity because the ferrous core is almost unaffected by the magnetic flux generated by the aluminum layers.

**Figure 5:**
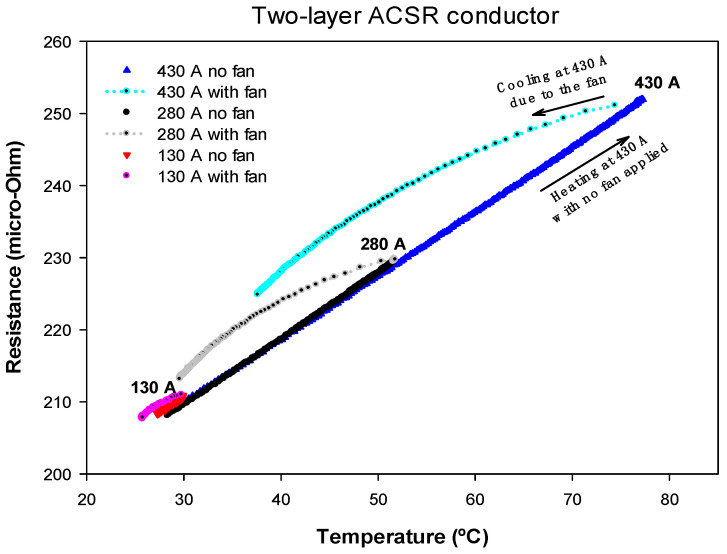
Two-layer ACSR conductor. *R_ac_* versus temperature measured for heating-cooling cycles at 430 A, 280 A and 130 A.

As in the previous case, the conductor was heated by applying the rated current until reaching the equilibrium temperature, which was about 77 °C. Then, once again, by forcing it to pass the same amount of current, the conductor was cooled using fans. During the course of the experiment, the conductor temperature, voltage drop and current were measured, so that the AC resistance was calculated from (4). This test was repeated with currents of 280 A and 130 A, where the results of the AC resistance versus the temperature of the conductor are displayed in Figure 5.

From Figure 5, it can be seen that, as expected, the AC resistance of the two-layer ACSR conductor depended on its temperature, but it was almost independent of the current level. The effect of a strong wind increased the *R_ac_* value below 5%.

### 4.3. Results Obtained with a Three-Layer ACSR Conductor

The third test was carried out with a three-layer conductor, whose rated current was around 1080 A. Due to the odd number of layers, the axial component of the magnetic flux does not completely cancel. Therefore, the AC resistance must depend slightly on the current intensity because the iron core is somewhat influenced by the axial component of the magnetic flux generated by the aluminum layers.

**Figure 6:**
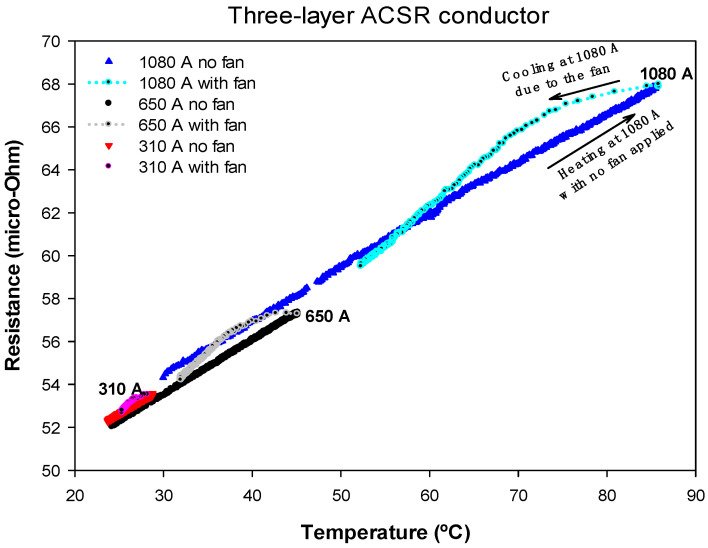
Three-layer ACSR conductor. *R_ac_* versus temperature measured for heating-cooling cycles at 1080 A, 650 A and 310 A.

The three-layer ACSR conductor was heated by applying the rated current until the equilibrium temperature was reached, which was about 85 °C. Next, by forcing the flow of the same amount of current, the conductor was cooled using fans. During the experiment, the temperature of the conductor, the voltage drop and the current were measured, so that the AC resistance was obtained from (4). The test was repeated twice with current intensities of 650 A and 310 A, respectively. Figure 6 shows the results of the AC resistance versus conductor temperature.

Results in Figure 6 show that the AC resistance of the three-layer ACSR conductor was almost independent of the current level due to the partial cancellation of the axial component of the magnetic flux due to the three layers of aluminum. The effect of a strong wind increased the AC resistance below 2.5%.

### 4.4. Results Summary

Table 2 shows the *R_ac_*_,0_ and *α* parameters obtained from a linear fit of the experimental data shown in Figure 4, Figure 5 and Figure 6 according to Equation (6), where *R*^2^ is the coefficient of determination of the linear regression.

Results presented in Table 2 clearly show that, in the case of the single-layer conductor, there was a step change in the AC resistance values *R_ac_*_,0_ measured at 20 °C for the three analyzed current levels. This was due to the effect of the axial component of the magnetic flux. However, for two- and three-layer conductors, *R_ac_*_,0_ was almost independent of the current level. Results presented in Table 2 also prove that the temperature coefficient of the resistance was almost independent of the current level and the topology of the conductor.

From the experimental results summarized in Table 2, and in the previous subsections, the following conclusions can be drawn:

- The AC resistance of two- and three-layer ACSR conductors was nearly independent of the current level, but this simplification cannot be applied to single-layer ACSR conductors. Therefore, for two- and three-layer ACSR conductors, it can be assumed that *R_ac_* = *R_ac_* (*T*), so that the heat gain due to the conductor losses *P_loss_* only depends on the conductor temperature, but not on the current level, i.e., *P_loss_* = *P_loss_* (*T*). In contrast, for single-layer conductors, *R_ac_* depends on both conductor temperature and current level, i.e., *R_ac_* = *R_ac_* (*T*,*I*), and hence *P_loss_* = *P_loss_* (*T*,*I*).
- In DLR applications, the conductor surface temperature is often measured, although it differs from the temperature of the internal strands. In strong wind conditions, the temperature difference between the surface of the conductor and the internal parts is typically greater. Therefore, in this study, for a given conductor surface temperature, the apparent AC resistance *R_ac_* measured in strong winds was larger than when measured without wind due to the increased radial temperature gradient under strong wind conditions. However, this difference was always below 5%, so it would not have a significant effect on the calculation of the DLR rating.

Therefore, based on the results obtained, two possible approaches are proposed below:

- Approach 1, which is valid for ACSR conductors with any number of layers. The current, conductor temperature, voltage drop and the phase shift between the voltage drop and the current must be measured, so that, by applying (4), the actual value of the AC resistance can be determined.
- Approach 2: Two- and three-layer ACSR conductors. For these conductors, the AC resistance *R_ac_* and thus, the heat gain due to conductor losses *P_loss_*, are almost independent of current level. Therefore, if the parameters *R_ac_*_,0_ and *α_ac_* are known, it is possible to measure only the current and the temperature of the conductor, thus avoiding the need to measure the voltage drop and the phase shift between the voltage drop and the current. This is advantageous because the voltage drop measurement has some drawbacks related to the addition of wires placed on the surface of the high-voltage ACSR conductors, with the consequent problems related to outdoor environments. Since *R_ac_* cannot be measured without measuring the voltage drop, if *R_ac_*_,0_ and *α_ac_* are known, *R_ac_* can be obtained by applying *R_ac,T_* = *R_ac_*_,0_[1 + *α_ac_*(*T* − *T*_0_)]. According to this equation, the temperature of the conductor, the parameters *R_ac_*_,0_ and *α_ac_* can be measured in the laboratory for a sample of the conductor, in a similar way as has been done in this paper.
- Approach 2: Single-layer conductor. In single-layer conductors, both the AC resistance *R_ac_* and the heat gain due to conductor losses *P_loss_*, depend on the current level and the temperature of the conductor. In this case it is also possible to avoid measuring the voltage drop. According to the values presented in Table 2, *α_ac_* can be considered as a constant value, so the current level determines *R_ac_*_,0_. Then, *R_ac_* can be obtained by applying *R_ac_*_,*T*_ = *R_ac_*_,0_[1 + *α_ac_*(*T* − *T*_0_)]. Once the values of the parameters *R_ac_*_,0_ and *α_ac_* summarized in Table 2 are known, they can be interpolated for any current level.

**Figure 7:**
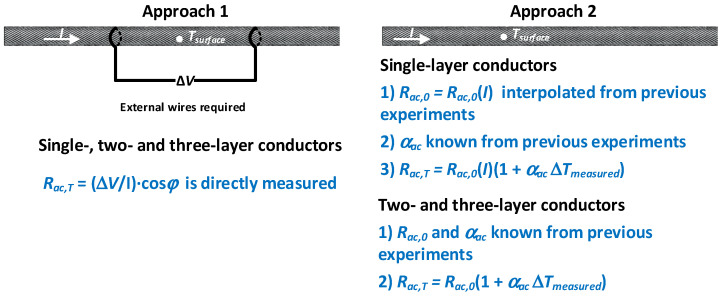
Proposed strategies to measure the AC resistance of the conductor as a function of the temperature and current level.

Figure 7 summarizes the two proposed strategies to measure the AC resistance of the conductor as a function of temperature and current level.

## 5. Conclusions

This paper has proposed two methods for the on-line determination of the AC resistance of aluminum conductor steel-reinforced (ACSR) conductors. The AC resistance is a key factor when applying dynamic line rating (DLR) approaches, as it determines Joule and core losses. This paper has analyzed the dependence of the AC resistance of ACSR with the line current, this being a novelty of this work, since most of the current DLR approaches do not consider this dependency, so they can lead to inaccurate estimates of the AC resistance, and thus of the DLR ampacity. To this end, single-layer, two-layer and three-layer conductors have been analyzed. Experimental results showed that while for two- and three-layer conductors the AC resistance is almost independent of the level of current passing through the conductors, for single-layer conductors, the AC resistance is influenced by the current level.

The first and more accurate method is based on a simultaneous measurement of the voltage drop along a certain length of the conductor, the current, the phase-shift between the voltage drop and the current, as well as the temperature of the conductor. However, this method has the disadvantage of requiring operators to install wires to measure the voltage drop. The second method avoids measuring the voltage drop and the phase shift, thus simplifying installation and measurement requirements, although it requires prior laboratory experiments to determine the required parameters *R_ac_*_,0_ and *α_ac_*.

The approaches presented in this paper are in line with the current need to operate power lines at their maximum instantaneous capacity by adapting in real-time the current rating to the existing weather conditions. These approaches are easy to implement, require low computational load and are compatible with IoT applications.

## Figures and Tables

**Table 1:** Main parameters of the three-layer 550-AL1/71-ST1A ACSR conductor from HAASE Gesellschaft and the two-layer 135-AL1/22-ST1A ACSR conductor from EMTA Kablo.

Symbol	Description	Three-Layer	Two-Layer	Unit
$A_{Al}$	Area of aluminum	549.7	134.9	mm^2^
$A_{steel}$	Area of steel	71.3	22	mm^2^
$N_{Al}$	Number of aluminum wires	54 (12/18/24)	26 (10/16)	-
$N_{Steel}$	Number of steel wires	7	7	-
$D_{Al}$	Aluminum wire diameter	3.6	2.57	mm
$D_{steel}$	Steel wire diameter	3.6	2.0	mm
*D*	Conductor diameter	32.4	16.3	mm
$m_{AL}$	Mass per unit length of aluminum	1.5183	-	kg/m
$m_{steel}$	Mass per unit length of steel	0.5583	-	kg/m
$R_{20{}^{\circ}C}$	DC resistance of the conductor	0.0526	0.2038	Ω/km
$I_{max}$	Current carrying capacity	1020	430	A

**Table 2:** Regression coefficients of (6).

Cable Type	Current	*R_ac_*_,0_	*α_ac_*	*R* ^2^
Single-layer	220 A	602.4 μΩ	0.0046 °C^−1^	0.9997
	145 A	535.2 μΩ	0.0048 °C^−1^	0.9991
	75 A	498.5 μΩ	0.0049 °C^−1^	0.9827
Two-layer	430 A	200.8 μΩ	0.0044 °C^−1^	0.9999
	280 A	200.2 μΩ	0.0046 °C^−1^	0.9996
	130 A	201.9 μΩ	0.0044 °C^−1^	0.9747
Three-layer	1080 A	52.3 μΩ	0.0046 °C^−1^	0.9987
	650 A	51.0 μΩ	0.0049 °C^−1^	0.9990
	310 A	51.4 μΩ	0.0047 °C^−1^	0.9843

## Data Availability

Not applicable.
